# Supplementary material for: Causes of Vaccine Hesitancy in Adults for the Influenza and COVID-19 Vaccines: A Systematic Literature Review
Source: Vaccines (Basel). 2022 Sep 13;10(9):1518. doi: 10.3390/vaccines10091518 (PMC9503596; doi:10.3390/vaccines10091518)
Supplement: Supplementary file 1 [file vaccines-10-01518-s001.zip › vaccines-1865662-supplementary.pdf]

**Table S1.** Search Strategy.

Search carried out in PubMed. Date of search: 27th October 2021.

| Searches | Number of hits |
|---|---|
| (Reasons) AND (vaccine hesitancy) AND (adult) | 143 |
| (Causes) AND (vaccine hesitancy) AND (adult) | 182 |
| (Reasons) AND (vaccine refusal) AND (adult) | 229 |
| (Causes) AND (vaccine refusal) AND (adult) | 204 |

**Table S2.** Data extraction table.

| Study | Country | Study design | Sample size | Type of vaccine | Population Focussed On | Causes of VH | Link to theme | Additional insights |
|---|---|---|---|---|---|---|---|---|
| Ahmed MAM, Colebunders R, Gele AA, et al. [6] | Somalia | Cross-sectional online survey | 4543 | COVID-19 | Adults (18 and above). Respondents were 62.4% male and more than 80% of them had been to university. The population was biased towards students. | Thought to be ineffective (6.22% respondents); scared of side effects (9.33% respondents); confidence in own immune system (4.49% respondents); belief that pandemic is over in Somalia (6.78% respondents). Females were less likely to accept the vaccine as they believed it could cause sterility and female gender was the only factor that was significantly associated with VH. The ability to procreate was stated as being a vital sign of womanhood in African settings. | Not needed Dangerous Cultural reasons | The fear that the vaccine could contain pork derived substances was identified by some participants as a reason for VH. |
| Ahmed TF, Ahmed A, Ahmed S, et al. [7] | Pakistan | Cross-sectional online survey | 655 after sample size calculation | COVID-19 | Adults aged 18 and over. Data was biased towards the 18-29 years age group. Those who were from rural areas and of a lower education background were underrepresented. | A Muslim's trust in God is enough protection- significantly associated with VH (AOR 2.45; 95% CI 1.34–4.48); the vaccine had not been tested sufficiently (AOR 2.75; 95%CI 1.67–4.53); the vaccine could harm those with low immunity (AOR 2.62; 95% CI 1.55–4.42); suffering from COVID-19 in the | Not needed Dangerous Lack of trust | Prompts from GPs did not seem to encourage participants to become vaccinated. The paper suggested religious leaders should be involved in creating communication strategies, especially to target lower income households. |

| | | | | | | | | |
|---|---|---|---|---|---|---|---|---|
| | | | | | | past was not associated with intention to become vaccinated, perhaps because many believed that having recovered from COVID resulted in lifelong immunity. | Cultural reasons | Those who were not following advice to combat COVID-19 were more unwilling to be vaccinated. There was a lack of trust in the government and in vaccines supplied from abroad which allowed opportunists to increase levels of VH in the population.<br>A successful vaccination campaign would investigate reasons causing negative views towards the vaccine. |
| Dorman C, Perera A, Condon C, et al. [8] | Orange County, USA | Online survey | 26,324 | COVID-19 | Adults aged 18 and over; those aged 75 and above were underrepresented. Women, those who were of non-Hispanic White ethnicity, those who were college educated, those who had English as their mother tongue and those who were 35-54 years of age were overrepresented. | Confidence in vaccination safety was a key determinant of willingness to be vaccinated ($r=0.723$, $p<0.001$). Concern for protecting others ($r=0.574$, $p<0.001$) and believing COVID-19 was serious enough to require vaccination ($r=0.478$, $p<0.001$) were also key determinants. All of these factors significantly predicted the willingness of the respondent to vaccinate themselves. | Not needed Dangerous | Overall, willingness to become vaccinated increased with age; race and ethnicity also impacted willingness to be vaccinated. Males were more willing to become vaccinated, as were Asian respondents. In addition, those who were the most and least educated were the most willing to receive vaccination. |
| El-Elimat T, AbuAlSamen MM, Almomani BA, et al. [9] | Jordan | Online cross-sectional survey | 3100 | COVID-19 | 18 years of age and above, mostly female, mostly undergraduates, most had health insurance and were without chronic disease | Independent factors predicting level of vaccine acceptance: If respondents believed in conspiracy theories, then they were less likely to accept vaccination (OR = 0.502, 95% CI = 0.356–0.709, $p < .001$). If they said that vaccination generally was safe, then they were nine times more likely to receive the vaccine (OR = 9.258, 95CI% = 6.020–14.237, $p < .001$). | Dangerous Lack of trust | 45.5% of respondents trusted HCPs as a source of information for vaccination, 30% trusted pharmaceutical companies, 17% trusted the internet and 16% trusted the media, 18.1% did not trust any source. One-third of participants in the study thought that vaccines made in Europe/America were the safest. |

| | | | | | | | | |
|---|---|---|---|---|---|---|---|
| | | | | | | Less than 60% of respondents believed that pharmaceutical companies would be able to make a safe and effective vaccination. 49.6% would not take the vaccine due to side effects. There was an 80% disapproval rate how the government were handling the pandemic in Jordan which related to increased VH. Many believed that the government would be unable to provide the vaccination for free. | | When asked about the least trusted sources of information social media and family members were stated. |
| Galistiani, G.F., Matuz, M., Matuszka, N. et al. [10] | Hungary | Cross-sectional study via an online questionnaire | 1631 | Influenza | The active adult population, aged 20-59 years | 47.4% believed that the vaccination could cause flu and 51.6% did not have enough knowledge about the safety of the ingredients of the vaccination.<br>55.4% of unvaccinated participants did not believe that an influenza vaccination was the best way to prevent influenza. | Not needed Dangerous | Factors associated with getting the vaccine were having a risk factor and social responsibility. Some said a HCP influenced them not to have the vaccination which may have been due to the HCP's own VH. Only one-third of those who had been vaccinated stated their decision was influenced by a HCP or family member. |
| Kreps S, Goldfarb J, Brownstein J, et al. [11] | USA | Cross-sectional Survey | 1027 | COVID-19 | 18 and above, nationally representative sample of US population | 63.9% of the hesitant group thought the side effects would be severe. Those who were not vaccine hesitant were significantly more likely to state that side effects were not severe than those who showed VH (50.5% vs. 27.5%; $p < 0.001$, two-tailed test). Those who had been vaccinated or who were willing to do so thought that the trial size was larger than those who were vaccine hesitant/ resistant- those who were not VH were 13% more likely to select the | Not needed Dangerous | Most participants significantly underestimated trial sizes for the Pfizer and Moderna trials and just over half knew that the vaccination does not contain live coronavirus. The study stated that vaccine willingness can be calculated by the perceived risk of the virus and the confidence and convenience of being vaccinated. |

| | | | | | | | | |
|---|---|---|---|---|---|---|---|---|
| | | | | | | largest trial size estimates than those who were unvaccinated (33.8% vs. 21.0%; difference in means is statistically significant, $p < 0.01$, two-tailed test). Those who were vaccinated or willing to receive the vaccination were significantly less likely to believe that the vaccine contained live coronavirus (54.5% vs. 35.3% $p < 0.001$, two-tailed test). | | |
| Kumari A, Ranjan P, Chopra S, et al. [12] | India | Thematic analysis of focus group discussions | 19 males, 24 females | COVID-19 | 18 years and over | Those who did not trust in the safety of vaccines were less likely to be vaccinated. Other reasons included a low number of COVID-19 cases; preferring natural immunity; being exposed to misinformation; fear of side effects; fear of receiving a fake vaccine; government conspiracies; concerns regarding safety and efficacy generally and for those who are elderly or with comorbidities and on children; believing the vaccine has been approved without enough data when compared to other vaccines; concern regarding rumours circulating social media or information from the news, family and friends; concern regarding unforeseen side effects; concern regarding the potential price of the vaccine and believing that the vaccine is not warranted due to the participant's age. | Not needed Dangerous Lack of trust | Those who showed a lower level of interest in, and a lower level of knowledge surrounding the vaccine may have exhibited this as they were dependent on less reliable sources of information. |

| | | | | | | | | |
|---|---|---|---|---|---|---|---|---|
| Montalti M, Di Valerio Z, Rallo F, et al. [13] | Italy | Cross-sectional survey | 443 | COVID-19 | Those aged over 18 years old from Bologna and Palermo from 5 pharmacies waiting to undergo a swab | Causes of vaccine hesitancy included a lack of trust in the vaccine as it had been newly developed (58.7% vs 55.5% Bologna vs Palermo); having seen information that made the participant wary of the vaccine (41.3% bs 26.7% Bologna vs Palermo); not wanting to be forced to take the vaccine (25.4% Bologna) and being aware of cases where people had become "damaged" as a result of vaccination (24.4% Palermo). | Not needed Danger ous Lack of trust | In Bologna, those who were willing to be vaccinated trusted that vaccines were safe and effective, they trusted healthcare professionals and had not had previous unpleasant reactions to vaccinations.<br>In Palermo, those who were vaccinated believed that vaccination was effective, had not had previous unpleasant experiences with vaccination and believed that COVID-19 infection prevented a higher level of risk than vaccination. |
| Quinn SC, Jamison A, Freimuth VS, et al. [14] | USA | Cross-sectional survey | 819 African American responden ts<br>838 White responden ts | Influenz a | Nationally representative sample of the USA, all were over 18 years of age | If participants perceived a higher level of racial fairness, then they were less likely to rely on naturalism and were more likely to agree on the moral obligation to be vaccinated for the health of others.<br>African American participants believed they was a higher risk of side effects; there was a lower risk of the flu and had a statistically significant higher dependency on naturalism as an alternative to vaccination.<br>Higher perceived racial fairness led to more trust in the vaccine and vaccine process and a more positive attitude overall. The frequency that participants experienced discrimination affected their perception of risk from the vaccine, their belief in conspiracy theories and it negatively affected the perceived | Not needed Danger ous Lack of trust | Overall, African American participants were more sceptical of the vaccine and vaccine process. This study found that racial factors had a higher influence on African American participants although discrimination was associated with VH in both of these groups and caused an increase in vaccine knowledge in African American participants but decreased levels of knowledge in White participants. |

| | | | | | | | | |
|---|---|---|---|---|---|---|---|---|
| | | | | | | moral obligation of being vaccinated for African American participants. | | |
| Qunaibi EA, Helmy M, Basheti I, et al. [15] | 23 Arab countries and territories and 122 other countries | Cross-sectional survey | 36,220 | COVID-19 | Arabs living in a total of 145 countries | Reasons for vaccine hesitancy included safety concerns(55.7%) and distrust in healthcare policies (39.1%), the production and testing of the vaccine (46.1%) and published studies. Many participants believed that vaccination was not needed as many people have already been infected and the infection rate is decreasing, the number of cases was significantly associated with vaccine acceptance. Around 1/5th of residents stated that the vaccine may not be effective against new strains. Other reasons included side effects being experienced , including effects that had not been disclosed (61.4%); that sufficient time has not passed to verify how safe the vaccine is; that production was rushed (46.1%); that the participant did not trust the published studies and/or the company making the vaccine (33%). There was a lower level of acceptance in those who had previously had either suspected or confirmed COVID-19 infection. If participants did not know which type of vaccine would be available to them, they were more likely to be vaccine hesitant (OR, 1.93), this could be because some Arab countries approved the Sinopharm | Not needed Dangerous Lack of trust | Participants from Arab countries were more likely to state that they did not believe the healthcare policies in their country, that there are no published studies on the vaccine and that they do not trust the company behind the vaccine or published studies. Some participants believed that the rate on infection was decreasing and those who felt that there was political interference in the pandemic and vaccination were more likely to be vaccine hesitant. Distrust also arose due to the inability of participants to choose which vaccine to be administered. VH was high is the vaccine type was to be unknown by the participant. As COVID severity is greater with older age, those aged 30-59 were less willing to be vaccinated. The COVID-19 case number (as opposed to the number of deaths) were associated with vaccine acceptance. |

| | | | | | | | | |
|---|---|---|---|---|---|---|---|---|
| | | | | | | vaccine without sufficient affirmative data. | | |
| Roy M, Sherrard L, Dubé È, et al. [16] | Canada | Cross-sectional survey | 108,700 | Influenza | Adults aged 18 and above. | Reasons for non-vaccination included that it was not necessary (66%-74%); a bad reaction to vaccination in the past (6%- 12%); a negative attitude towards the vaccine, especially in relation to its effectiveness; fear of being vaccinated (4%- 5%); believing the participant's health is excellent; not believing influenza to be a severe illness; believing there were low benefits of vaccination and believing the participant was not susceptible to influenza. | Not needed Dangerous Lack of trust | Perceived vulnerability to infection was shown to play a role in VH. Interestingly, as the vaccine was not provided for free, it could have been viewed as less necessary by some participants. |
| Subramaniam N, Patil S, Umakanthan S, et al. [17] | India | Population-based longitudinal survey | 3000 | COVID-19 | People aged 18 years and over | Participants who thought that COVID-19 precautions were unnecessary (8.1% of vaccine resistant respondents) and that the severity of COVID was being exaggerated were more likely to be vaccine hesitant but those who appreciated the healthcare system (11.1%) and the government's policies (13%) to fight COVID-19 were less likely to be vaccine hesitant.<br>88% of participants feared side-effects which included both minor and life-threatening side effects. 65% of participants stated that they felt that they did not have enough information about the vaccination.<br>Another issue was rumours circulated on social media regarding the vaccine | Not needed Dangerous Lack of trust | 61.2% of vaccine hesitant and resistant participants reconsidered or even changed their mind on vaccination following a six-month period in which they had access to more information on the expected side-effects of the vaccine and on vaccine safety. Those who were vaccine hesitant were not unwilling to seek out information regarding the safety and efficacy of vaccines from reliable sources.<br>Younger people who know that they are unlikely to experience severe disease often denied the vaccination as did those who did not approve of the government's handling of the pandemic. |

| | | | | | | | | |
|---|---|---|---|---|---|---|---|---|
| | | | | | | especially in vaccine resistant participants (87.6%). | | |
| Syed Alwi, S.A.R., Rafidah, E., Zurraini, A. et al. [18] | Malaysia | Cross-sectional survey | 1411 | COVID-19 | Malaysians aged 18 years and over | Reasons for hesitancy in VH respondents included fear of side effects (95.8%, RII= 0.98), fear of the level of safety of the vaccine (84.7%, RII= 0.92), lack of information surrounding the vaccine (80.9%, RII=0.90) and concerns on the effectiveness of the vaccine (63.6%, RII=0.82).<br>Some participants did not believe COVID to be dangerous (22.5%) and some were unwilling to be vaccinated as they believed in traditional remedies (17.8%) , or they had cultural (6.8%) or religious (20.8%) reasons not to be vaccinated.<br>Those who believed COVID-19 was a threat to health and that they were susceptible to infection were more likely to be vaccinated and those who did not trust healthcare professionals, or the government were unlikely to be vaccinated. | Not needed<br>Dangerous<br>Lack of trust<br>Cultural reasons | Participants' confidence in the vaccine's effectiveness could be affected by the level of risk that they associated with the vaccination. 80.9% of participants felt that they did not have sufficient information regarding the vaccine despite the fact that information was regularly aired across many channels including the radio and television. Participants may have felt too overwhelmed with the level of information available. |
| Wong K, Cohen A, Norris S, et al. [19] | Soweto and Klerksdorp, South Africa | Cross-sectional survey | 973 households in Soweto and 1,442 in Klerksdorp | Influenza | Primary caregivers aged 18 years and over | Many participants believed that the influenza vaccine would not prevent influenza (Soweto: 23, 19%; Klerksdorp: 17, 19%; P = 0.9) and some participants stated that they would not like to become vaccinated as they did not have enough information surrounding the vaccine (Soweto: 10, | Not needed<br>Dangerous<br>Lack of trust | Many participants did not believe that it was possible to die due to influenza and there was not a good level of understanding of the purpose of vaccination. |

| | | | |
|---|---|---|---|
| | | 8%; Klerksdorp: 9, 10%). Participants were more likely to become vaccinated if a member of the household was HIV-positive but were less likely to be vaccinated if they believed it was not safe. Some participants stated that they did not want to become vaccinated as they had taken the vaccine before and had become sick; because it must be taken yearly; because they believe in natural remedies, God, and prayer and because they do not want to become habituated to it.<br>Other reasons included that the vaccine being free of charge did not guarantee its effectiveness; that the flu is natural, but the vaccine contains drugs and that they did not believe it would work. | Cultural reasons |
